# EPIDEMIOLOGY OF TRAUMATIC FRACTURES OF THE SPINE IN MARIO COVAS HOSPITAL BETWEEN 2015 AND 2020

**DOI:** 10.1590/1413-785220233103e267571

**Published:** 2023-09-08

**Authors:** Cássio Bousada Franco, Pedro Henrique Swinerd Coelho da Cruz, Caio Cesar Lucchese Moreno, Igor Oliveira Meneses, Lara Guercio dos Santos, Antonio Carlos Fontoura, Thiago Kolachinski Brandão, Luciano Miller Reis Rodríguez, Adriano Masayuki Yonezaki

**Affiliations:** 1 Instituto de Assistência Médica ao Servidor Público Estadual de S. Paulo /IAMSPE, São Paulo, São Paulo, Brazil.; 2 Faculdade de Medicina do ABC, , São Paulo, Brazil.; 3 Hospital da Alvorada, São Paulo, São Paulo, Brazil.; 4 Hospital Israelita Albert Einstein, São Paulo, São Paulo, Brazil.

**Keywords:** Fracture, Spine, Trauma, Coluna Vertebral, Fratura, Trauma

## Abstract

**Objectives:**

Analyze the epidemiological profile of patients with traumatic spinal fractures treated at Mário Covas State Hospital between 2015 and 2020.

**Methodology:**

This is an epidemiological, descriptive, retrospective, quantitative, comparative, medical records review-type study. Data collection was carried out between May and June 2022 at the Mário Covas State Hospital, the following characteristics being evaluated: age, sex, lesion topography, trauma mechanism, origin and treatment.

**Results:**

Data from 252 patients with traumatic spinal fractures were analyzed. The mean age of patients was 48.7 years, 74.7% were male. The mechanism of trauma from falls from a height and the topography of the lumbar vertebrae have a highly significant trend. The most affected vertebrae are lumbar L1, thoracic T12 and cervical C6. The crossing of the age group with the male sex is higher than expected in those over 60 years of age. The crossing of the age group with the trauma mechanism is higher than expected, between 20 and 39 years.

**Conclusion:**

There are few published works on the epidemiology of traumatic fractures of the spine, which points to the need for further studies on the subject. Level of Evidence III; Retrospective comparative study.

## RESUMO

**Objetivos:**

Analisar o perfil epidemiológico dos pacientes com fratura traumática de coluna vertebral atendidos no Hospital Estadual Mário Covas entre os anos de 2015 e 2020.

**Metodologia:**

Trata-se de um estudo epidemiológico, descritivo, retrospectivo, quantitativo, comparativo, do tipo revisão de prontuário médico. A coleta de dados foi realizada entre maio e junho de 2022 no Hospital Estadual Mário Covas, sendo avaliadas as características: idade, sexo, topografia da lesão, mecanismo de trauma, procedência e tratamento.

**Resultados:**

Foram analisados dados de 252 pacientes com fratura traumática da coluna vertebral. A média da idade dos pacientes foi de 48,7 anos, 74,7% eram do sexo masculino. O mecanismo de trauma tipo queda de altura e a topografia das vértebras lombares têm tendência altamente significativa. As vértebras mais afetadas são lombar L1, torácica T12 e cervical C6. O cruzamento da faixa etária com sexo masculino está acima do esperado nos maiores de 60 anos. O cruzamento da faixa etária com mecanismo do trauma está acima do esperado, entre 20 a 39 anos.

**Conclusão:**

São poucos os trabalhos publicados a respeito da epidemiologia das fraturas traumáticas de coluna vertebral, o que aponta para a necessidade de novos estudos acerca da temática. Nível de evidência III; Estudo retrospectivo comparativo.

## INTRODUCTION

Currently, traumatic spinal fractures are important causes of morbidity and mortality and represent a gradual increase in incidence. Thus, it is notable the growth in the number of patients who arrive at the emergency room with severe traumatic spinal injuries, victims of falls from heights, automobile accidents, firearm wounds, and being run over by a car.^1^

Falling from a balcony has been found to be the most frequent cause among trauma mechanisms associated with traumatic fracture of the spine. Housing with balconies is related to the shift in construction to masonry houses in large population areas and pockets of poverty. Given its correlation with the state of social vulnerability of the individual, it is understood as a modifiable causal factor.^2^

Traumatic spine injury occurs predominantly in males, and is four times more frequent in the 15-40 age group,^3^ that is, at an age of high productivity. Given the risk of irreversible sequelae, the consequences affect not only the patient, but also the family and society, and thus have a major impact on public health in Brazil.^2^

It is emphasized that few studies have been published in the Brazilian literature on the epidemiology of traumatic spine fractures, which justifies the need for new studies on the subject. In this sense, recognizing the scarcity of such data, this study aims to analyze the epidemiological profile of patients with traumatic spine fractures treated at the Mário Covas State Hospital between 2015 and 2020.

Thus, the intention is, based on the results, to evaluate possible techniques for improving treatment, as well as prevention strategies. In addition to analyzing the impact of the COVID-19 pandemic on the incidence of spinal trauma during the years 2020 in relation previous years, disseminating these data as a form of scientific contribution.

## METHODS

This is an epidemiological analysis, descriptive, retrospective, quantitative, comparative study, with a direct and observational approach, of the medical record review type. Data collection was performed by analyzing clinical and epidemiological data of patients seen at Hospital Estadual Mário Covas (HEMC), during the months of May and June 2022. Thus, the characteristics evaluated were age, gender, topography of the injury, trauma mechanism, origin, and treatment. It is noteworthy that the HEMC is the trauma reference center of the ABC Paulista macro region, receiving patients regulated from other cities in greater São Paulo, through the CRUE (Central Regulatory Urgency and Emergency).

The data studied were divided into groups: year of attendance, age group, sex, trauma mechanism (auto accident, motorcycle accident, domestic accident, auto vs. motorcycle accident, running over, FAF, diving, fall, and fall from own height), topography (odontoid, cervical/odontoid vertebra, lumbar vertebra, thoracic vertebra, thoracic/cervical vertebra, thoracic/cervical/odontoid vertebra, thoracic/lumbar vertebra, thoracic/lumbar/cervical vertebra, others). Surgical and conservative management was also addressed.

The data were expressed with their respective confidence intervals, and the statistical treatment was performed using SPSS software. For comparison of proportions, the chi-square test was used with a 5% significance level and, finally, the variables: age group and gender, cause and gender, cause and age group.^4^

Regarding data collection from medical records, criteria were established. As inclusion criteria, patients with a diagnosis of traumatic spine fracture were selected for this study, having undergone surgical treatment or not, performed at the HEMC during the period from 2015 to 2020, as these were the years with the highest prevalence of medical care. The exclusion criteria were non-traumatic fractures and patients about whom it was not possible to collect adequate information by studying the medical records.

## RESULTS

Data from n=252 patients with traumatic spine fracture from 2015 to 2020 were analyzed. Patients were aged between 9 years (youngest) and 91 years (oldest), mean age is 48.7 ±18 years, median = 50 years (34 to 62, 1st Quartile and 3rd Quartile). The age group 40 to 59 years (34.9%) was significantly more frequent. Of the patients are 74.7% were male (p-value <0.0001*, statistically significant).

To facilitate the inspection of the results, Figures 1 to 8 were elaborated, which cover the year, age range, gender, trauma mechanism, topography, cervical vertebrae and thoracic vertebrae of the patients with traumatic fractures, respectively.

**Figure 1:**
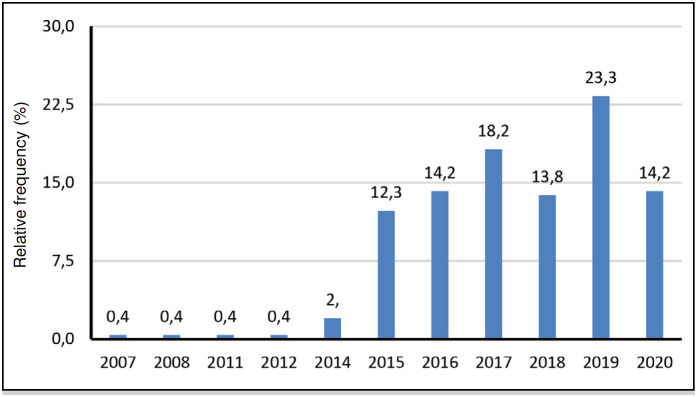
Year of the traumatic spine fractures of 252 patients.

**Figure 2:**
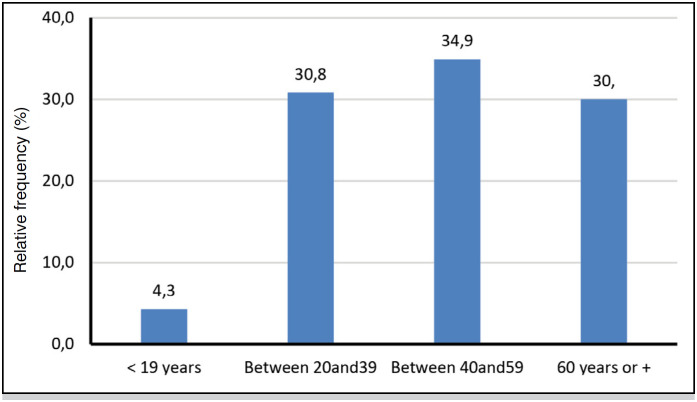
Age range of the 252 patients with traumatic spinal fracture.

**Figure 3:**
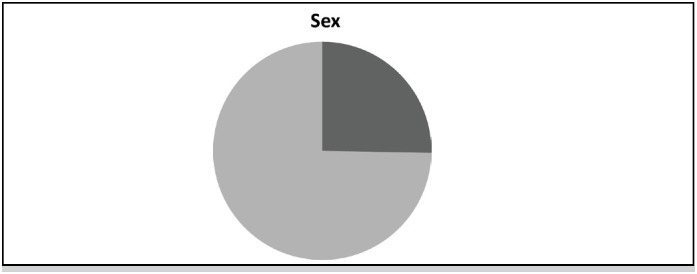
Sex of the 252 patients with traumatic spinal fracture.

**Figure 4:**
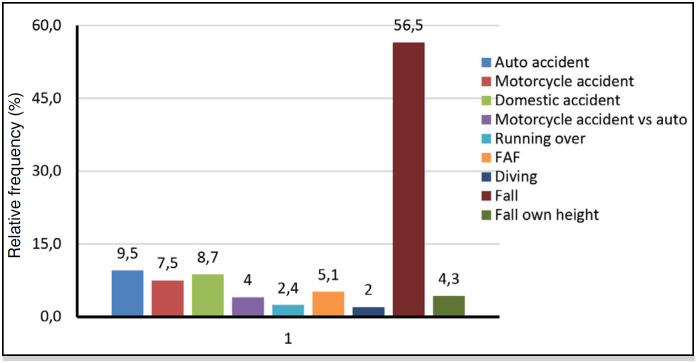
Mechanism of trauma of the 252 patients with traumatic spinal fracture.

**Figure 5:**
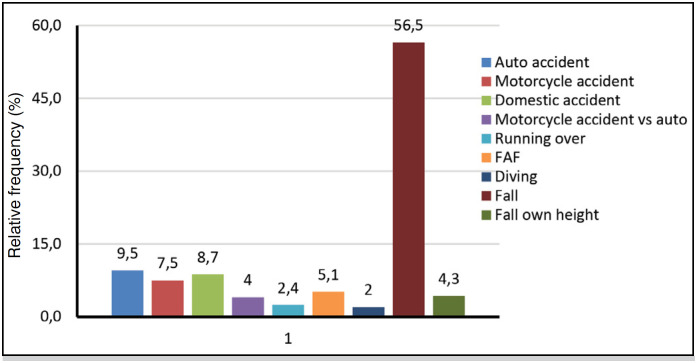
Topography of the 252 patients with traumatic spinal fracture.

**Figure 6:**
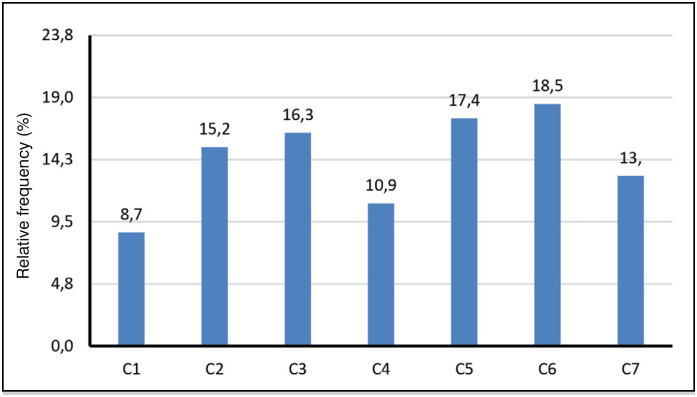
Affected cervical vertebrae of the 252 patients with traumatic spinal fracture.

**Figure 7:**
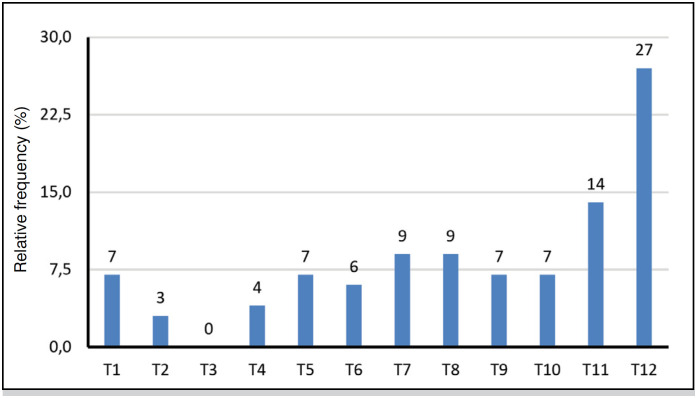
Thoracic vertebrae affected in the 252 patients with traumatic spinal fracture.

**Figure 8:**
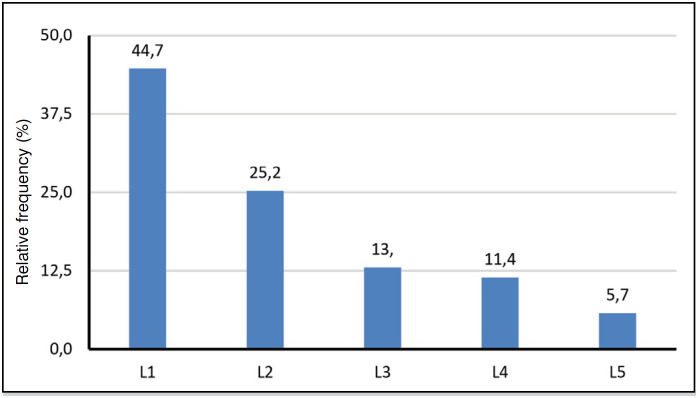
Affected lumbar vertebrae of n=252 patients with traumatic spinal fracture.

Table 1 shows the intersection of the age range data with the following variables:

- Sex: male (57.9%) is above expected in the 60 years and older age group, (p-value=0.0006*, highly significant).
- Mechanism of trauma: above expected in the 20 to 39 age group: motorcycle accident (17.9%), motorcycle vs auto accident (11.5%), FAF (14.1%).
- Conduct: Surgical (52.3%) in the 40 to 59 age group, the most prevalent.

**Table 1:** Evaluation of age group according to sex, trauma mechanism, management and follow-up of n=252 patients with traumatic spine fracture.

	< 19 years		20 e 39		40 e 59		60 years or +		
	n=11	%	n=78	%	n=88	%	n=76	%	p-value
**Sex**									**0.0006** *
Feminine	2	18,2	11	14,1	19	21,6	32 (z)	42,1 (z)	
Masculine	9	81,8	67	85,9	69	78,4	44 (x)	57,9 (x)	
**Trauma mechanism**								**<0.0001** *
Auto accident	2	18,2	6	7,7	9	10,2	7	9,2	
Motorcycle accident	1	9,1	14 (x)	17,9 (x)	2 (z)	2,3 (z)	2	2,6	
Domestic accident	2	18,2	5	6,4	9	10,2	6	7,9	
Motorcycle accident vs auto	0	0,0	9 (x)	11,5 (x)	1	1,1	0 (z)	0,0 (z)	
Running over	1	9,1	0	0,0	2	2,3	3	3,9	
FAF	1	9,1	11 (x)	14,1 (x)	0 (z)	0,0 (z)	1	1,3	
Diving	0	0,0	3	3,8	2	2,3	0	0,0	
Fall	4	36,4	29 (z)	37,3 (z)	61 (x)	69,3 (x)	49	64,6	
Fall own height	0	0,0	1	1,3	2	2,3	8	10,5	
**Conduct**									**0,4897**
Arthrodesis	4	36,4	39	50,0	46	52,3	32	42,1	
Conservative	7	63,6	39	50,0	42	47,7	44	57,9	

* Independence chi-square.

(x) Above expectations.

(z) Lower than expected.

Table 2 shows that the intersection of the data on the trauma mechanism in relation to gender, with motorcycle accidents being the most prevalent in males (10.1%) and falls from height in females (10.9%).

**Table 2:** Evaluation of gender according to trauma mechanism and management of n=252 patients with traumatic fracture of the spine.

	Feminine		Masculine		
	n=64	%	n=189	%	p-value
**Trauma mechanism**					**<0.0001** *
Auto accident	9	14,1	15	7,9	
Motorcycle accident	0	0,0	19	10,1	
Domestic accident	3	4,7	19	10,1	
Motorcycle accident vs auto	0	0,0	10	5,3	
Running over	1	1,6	5	2,6	
FAF	2	3,1	11	5,8	
Diving	0	0,0	5	2,6	
Fall	42	65,6	101	53,5	
Fall own height	7	10,9	4	2,1	
**Conduct**					**0,3681** *
Arthrodesis	27	42,2	94	49,7	
Conservative	37	57,8	95	50,3	

* Independence chi-square.

Table 3 shows that there is no statistical difference between the trauma mechanism and the type of treatment adopted.

**Table 3:** Evaluation of Management according to Mechanism of trauma and of n=252 patients with traumatic fracture of the spine.

Conduct	Arthrodesis		Conservative		
	n=121	%	n=132	%	
Auto accident	12	9,9	12	9,1	
Motorcycle accident	9	7,4	10	7,6	
Domestic accident	12	9,9	10	7,6	
Motorcycle accident vs auto	6	5,0	4	3,0	
Running over	3	2,5	3	2,3	
FAF	5	4,1	8	6,1	
Diving	2	1,7	3	2,3	
Fall	68	56,2	75	56,7	
Fall own height	4	3,3	7	5,3	
p-value					0,9738 *

* Independence chi-square.

Table 4 shows that the trauma mechanism automobile accident (19.5%) and diving (7.9%) are common causes of cervical vertebra fracture, but falls are more prevalent in both cervical (42.9%) and lumbar vertebra fractures (69.3%).

**Table 4:** Evaluation of Topography according to Mechanism of trauma and of n=252 patients with traumatic spinal fracture.

Topography *	Odontoid		Cervical		Cervical/Odontoid		Lumbar	
	n=6	%	n=63	%	n=2	%	n=101	%
Auto accident	1	16,7	10 (x)	15,9 (x)	0	0,0	3	3,0
Motorcycle accident	1	16,7	4	6,3	0	0,0	6	5,9
Domestic accident	1	16,7	7	11,1	1 (x)	50,0 (x)	7	6,9
Motorcycle accident vs auto	0	0,0	2	3,2	0	0,0	4	4,0
Running over	0	0,0	2	3,2	0	0,0	3	3,0
FAF	0	0,0	4	6,3	0	0,0	5	5,0
Diving	0	0,0	5 (x)	7,9 (x)	0	0,0	0	0,0
Fall	3	50,0	27	42,9	1	50,0	70 (x)	69,3 (x)
Fall own height	0	0,0	2	3,2	0	0,0	3	3,0

* p-value <0.0001. Chi-square of independence.

(x) Above expectations.

Table 5 shows that as with lumbar vertebral fractures, the trauma mechanism of falling is the most common in thoracic vertebral fractures (52.8%).

**Table 5:** Evaluation of Topography according to Mechanism of trauma and of n=252 patients with traumatic spine fracture.

Topography	Thoracic		Thoracic/Cervical		Thoracic/Cervical/Odontoid	
	n=53	%	n=7	%	n=1	%
Auto accident	5	9,4	2	28,6	1	100,0
Motorcycle accident	7	13,2	0	0,0	0	0,0
Domestic accident	3	5,7	0	0,0	0	0,0
Motorcycle accident vs auto	3	5,7	1	14,3	0	0,0
Running over	0	0,0	1	14,3	0	0,0
FAF	2	3,8	1	14,3	0	0,0
Diving	0	0,0	0	0,0	0	0,0
Fall	28 (x)	52,8 (x)	1	14,3	0	0,0
Fall own height	5	9,4	1	14,3	0	0,0

* p-value <0.0001. Chi-square of independence.

(x) Above expectations.

Table 6 shows that most fractures of lumbar vertebrae were treated surgically, while fractures of the odontoid process were almost entirely treated conservatively.

**Table 6:** Evaluation of Management according to Topography of n=252 patients with traumatic spine fractures.

Conduct	Surgical		Conservative	
	n=121	%	n=132	%
**Topography** *				
Odontoid	1	0,8	5	3,8
Cervical	28	23,1	35	26,5
Cervical/Odontoid	1	0,8	1	0,8
Lumbar	49	40,6	52	39,3
Thoracic	29	24,0	24	18,2
Thoracic /Cervical	5	4,1	2	1,5
Thoracic /Cervical/Odontoid	0	0,0	1	0,8
Thoracic /Lumbar	3	2,5	6	4,5
Thoracic /Lumbar/Cervical	0	0,0	1	0,8
Sacrum	5	4,1	5	3,8

* p-value: 0.5517. Chi-square of independence.

Therefore, from the statistical analysis performed by means of the information collected from the medical records regarding spinal fractures that occurred at the HEMC in the aforementioned study period, the following predominant characteristics can be stated: mean age is 48.7 +/- 18 years, age range 40 to 59 years (34.9%) is the most frequent, and 74.7% are male. The trauma mechanism of falling from height and lumbar vertebrae topography (39.8%) have a highly significant trend.

The most affected vertebrae are: lumbar L1 (44.7%), followed by thoracic T12 (27.0%) and cervical C6 (18.5%). The crossover of age group with male gender (57.9%) is higher than expected in the 60 and older age group. The intersection of age with the trauma mechanism is above what is expected in the 20 to 39 age group: motorcycle accident (17.9%), motorcycle vs auto accident (11.5%), gunshot wound (14.1%) and in the 40 to 59 age group, fall (69.3%).

Figure 1 shows a drop in the incidence of traumatic spine fractures between 2019 and 2020, which corresponds to the period when the new coronavirus pandemic begins. It is also noted that conservative management (52.2%) was the most common approach to spinal fractures, but without a large percentage of difference with respect to surgical management (47.8%).

## DISCUSSION

An increase in the number of patients who are victims of spinal trauma has been noted, with a significant socioeconomic impact.^5^ The male gender with the highest prevalence has also been observed by other authors, which corroborates the findings of this study. Regarding the age range, the age of higher prevalence was around 40 years, varying in most cases from 20 to 60 years of age,^3 , 5 – 10^ being an age range close to the patients seen at the HEMC.

It is noteworthy that, besides trauma from falls from heights, which are very prevalent,^5^ traffic accidents deserve attention, since its high prevalence has been observed, as well as the involvement of victims in an even younger age range when compared, reaching a decade less age in traffic accidents when compared to the trauma caused by this type of fall mentioned above.^6^

Other studies have shown the presence of different etiologies of spinal injury by trauma, such as automobile accidents (25%-50%), falls from a slab (20-23%), firearm wounds (7%), diving in shallow waters (3%), sports practice, aggression (2%) and other acts of violence (15%).^5 – 7^ The causes vary according to the region studied, in view of the percentages of these indices contrasting with those found in patients seen at the HEMC.

Furthermore, it was verified that the lumbar spine L1 is also usually the most affected, with the thoracic spine T12 having a relevant prevalence.^6^ In females, a greater prevalence of the cervical spine was verified, at a ratio of 6:1 in relation to males, even though the latter are the ones who suffer more spinal injuries as a result of traumatic events.^7^

In a study carried out in Saudi Arabia, the prevalence of cervical injuries was also higher in males, with 85.6% of the cases and a mean age of 36.6 years.^9^

No less important is the presence of neurological injury as a result of the trauma.^6^ A European study observed that among the patients assisted, (9.6%) suffered spinal fractures/dislocations alone and 4,489 (1.8%) suffered spinal cord injury with or without fractures/dislocations. The age of patients with spinal cord injury was 44.5 years, and 64.5% of these patients were male.^10^

However, even with these important indexes, the incidence of spinal cord injury due to spinal trauma is not elucidated in Brazil.^7^ In this sense, the understanding of these data, as well as in relation to other types of spinal injuries, is essential for the planning of health services and for the establishment of injury prevention priorities.^11^

With the high prevalence of spinal injuries, improvements in management were required to achieve effective treatment. Thus, the development and training of teams specialized in the care of these patients was expanded, in order to provide a greater expectation of survival, even in the most severe cases, in addition to reducing complications. However, the prognosis depends on the rehabilitation process to reintegrate the individual into society, which is a long process, and emphasizes the importance of prevention.^7^

It is also emphasized that with the drop in traumatic spinal fractures in 2020 mentioned in Figure 1 , it is assumed that this fact may be related to social isolation due to the pandemic of COVID-19, in view of the growth in incidence in relation to the years before the onset of the global health crisis.

## CONCLUSIONS

Thus, traumatic spinal injuries commonly affect young adults and males. In addition, fractures of the lumbar and thoracic spine have become more frequent. It is noteworthy that the epidemiology, etiology, and mechanism of injury can vary according to the location studied, with high-impact traffic accidents and occupational accidents being an important cause. Besides the physical and financial incapacitation, spinal injuries interfere with the patient's quality of life.

However, there are still few studies published on the epidemiology of traumatic spine fractures, and therefore, we suggest the need for further research on the subject for better knowledge and planning of necessary interventions.
